# Dapagliflozin Ameliorates Cognitive Impairment in Aluminum-Chloride-Induced Alzheimer’s Disease via Modulation of AMPK/mTOR, Oxidative Stress and Glucose Metabolism

**DOI:** 10.3390/ph16050753

**Published:** 2023-05-16

**Authors:** Waad A. Samman, Salma M. Selim, Hassan M. El Fayoumi, Norhan M. El-Sayed, Eman T. Mehanna, Reem M. Hazem

**Affiliations:** 1 Department of Pharmacology and Toxicology, College of Pharmacy, Taibah University, Medina 30078, Saudi Arabia; 2 Department of Pharmacology and Toxicology, Faculty of Dentistry, Sinai University, Kantara, Ismailia 41636, Egypt; 3 Department of Pharmacology and Toxicology, Faculty of Pharmacy, Suez Canal University, Ismailia 41522, Egypt; 4 Department of Biochemistry, Faculty of Pharmacy, Suez Canal University, Ismailia 41522, Egypt

**Keywords:** Alzheimer’s disease, dapagliflozin, AMPK pathway, oxidative stress, glucose metabolism

## Abstract

Alzheimer’s disease (AD) is a progressive neurological illness characterized by memory loss and cognitive deterioration. Dapagliflozin was suggested to attenuate the memory impairment associated with AD; however, its mechanisms were not fully elucidated. This study aims to examine the possible mechanisms of the neuroprotective effects of dapagliflozin against aluminum chloride (AlCl_3_)-induced AD. Rats were distributed into four groups: group 1 received saline, group 2 received AlCl_3_ (70 mg/kg) daily for 9 weeks, and groups 3 and 4 were administered AlCl_3_ (70 mg/kg) daily for 5 weeks. Dapagliflozin (1 mg/kg) and dapagliflozin (5 mg/kg) were then given daily with AlCl_3_ for another 4 weeks. Two behavioral experiments were performed: the Morris Water Maze (MWM) and the Y-maze spontaneous alternation (Y-maze) task. Histopathological alterations in the brain, as well as changes in acetylcholinesterase (AChE) and amyloid β (Aβ) peptide activities and oxidative stress (OS) markers, were all evaluated. A western blot analysis was used for the detection of phosphorylated 5’ AMP-activated protein kinase (p-AMPK), phosphorylated mammalian target of Rapamycin (p-mTOR) and heme oxygenase-1 (HO-1). Tissue samples were collected for the isolation of glucose transporters (GLUTs) and glycolytic enzymes using PCR analysis, and brain glucose levels were also measured. The current data demonstrate that dapagliflozin represents a possible approach to combat AlCl_3_-induced AD in rats through inhibiting oxidative stress, enhancing glucose metabolism and activating AMPK signaling.

## 1. Introduction

Alzheimer’s disease (AD) is a serious neurological illness that causes cognitive decline and memory loss due to the destruction of neurons in the hippocampus and cortex [1]. By 2050, 106.8 million AD patients are expected to exist worldwide, making it a growing public health concern with substantial socioeconomic implications [1]. Most cases of AD commonly manifest in patients over 65 years old (late-onset AD), with some cases occurring in patients as young as their mid-40s or early 50s (early-onset AD) [2]. This disorder is described by the production and buildup of amyloid β (Aβ) plaques and tau amyloid fibrils, which lead to synapse loss and neurodegeneration [3].

Many theories have been proposed in AD, such as the cholinergic theory, the amyloid hypothesis and the tau hypothesis [4]. It has been shown that significant changes occur in the cholinergic system of the brain, which is correlated with cognitive function [5]. Acetylcholinesterase (AChE) is aggregated in senile plaques with Aβ accumulation. In addition, they induce the production of hazardous Aβ-AChE complexes that are more harmful than amyloid fibrils [6]. It causes difficulties recalling spatial sequences, attacks emotional responses and affects learning and memory as a result of its effects [7]. As a result, elevated Aβ promotes other disease-related features, such as hyper-phosphorylation of tau and the presence of neurofibrillary tangles (NFT) [8].

There is evidence that oxidative stress (OS) plays a role in AD [9] because the brain is especially vulnerable to reactive oxygen species (ROS). Furthermore, the brain lacks a robust antioxidant system and contains fatty acids, which increase peroxidation. It also uses a large amount of oxygen, making it more susceptible to free radicals [10]. The neurotoxic effects of Aβ are related to OS, which causes damage to membranes, lipids, proteins and nucleic acids in neurons by generating ROS [11].

Aluminum (Al) is a potent neurotoxin that has been linked to a variety of neurodegenerative diseases, including AD and dementia [12]. Aluminum passes across the blood–brain barrier (BBB) via high-affinity receptors [13]. Our bodies are being exposed to higher levels of Al as a consequence of high-rate global industrialization, resulting in pollution of the air, water, and even drugs [14]. Excessive Al consumption may cause Aβ deposits in central nervous cells and increase the amount of amyloid precursor protein (APP), making it a possible risk factor for AD [15].

The brain utilizes glucose as the main energy source for its neurons and astrocytes [16]. However, in AD, neurons become unable to raise their energy production through glycolysis due to the significant decrease of the glycolytic enzymes. This deficiency of sufficient energy can trigger the upregulation of oxidative phosphorylation that further contributes to OS and mitochondrial dysfunction, leading to neuronal loss and dementia [16].

The antidiabetic, sodium-glucose cotransporter 2 inhibitors (SGLT2is) prevent glucose and salt resorption in the kidneys [17]. Dapagliflozin, a SGLT2i, was demonstrated to have insulin-independent glucose reduction with a little risk of hypoglycemia, resulting in enhanced urinary glucose excretion [18]. It has been proved that SGLT2is improve cognitive function in obese and type 2 diabetic mice. The mechanism of this SGLT2i beneficial effect on cognition was associated with reduced cerebral OS [19]. As a result, there is mounting evidence that SGLT2is have neuroprotective potential [20]. Second, our study highlighted the effect of dapagliflozin on glucose transporters and glycolytic enzymes, which was an additional mechanism to its neuroprotective effect reported before. The previous study [21] focused on the effect of dapagliflozin as an autophagy enhancer.

As mentioned above, the goal of this research was to assess the potential protective benefits of dapagliflozin against AlCl_3_-induced AD in rats. The current study investigates the possible impact of dapagliflozin on the cholinergic system, glucose metabolism, AMPK/mTOR and OS in AD, clarifying the underlying mechanism of the alleged neuroprotective action of dapagliflozin.

## 2. Results

### 2.1. Dapagliflozin Improved Acquisition Impairment and Depressive Behavior

#### 2.1.1. Dapagliflozin Effect on Morris Water Maze

**Figure 1:**
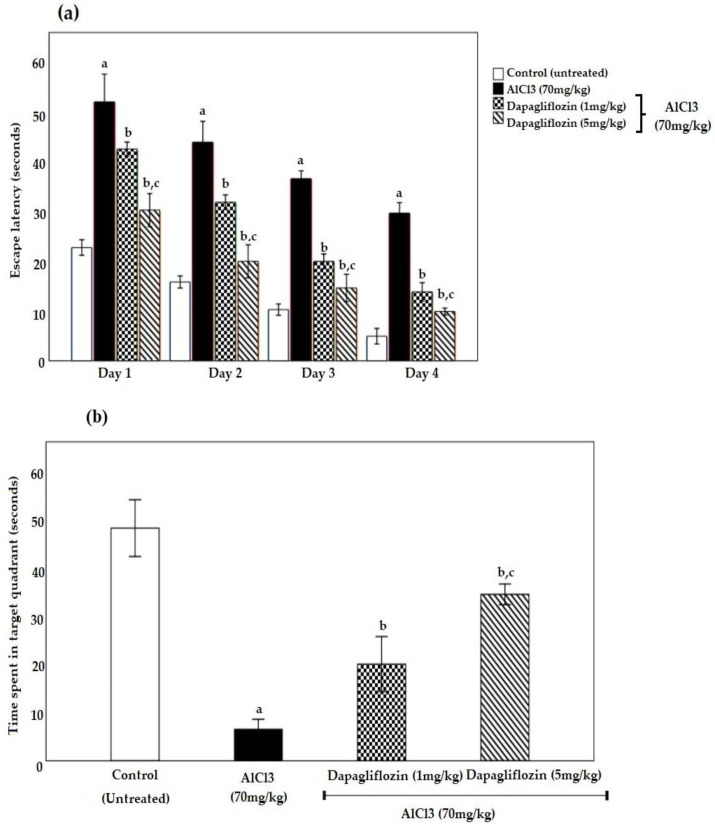
Effect of dapagliflozin (1 and 5 mg/kg) on (**a**) escape latency (seconds) and (**b**) time spent in target quadrant (seconds) during the induction of AD. Data expressed as mean ± standard deviation and analyzed using two-way ANOVA followed by Tukey’s comparison tests. a: significant versus control group, b: significant versus AlCl_3_ (70 mg/kg), c: significant versus AlCl_3_ (70 mg/kg) + dapagliflozin (1 mg/kg). AD: Alzheimer’s disease, AlCl_3_: aluminum chloride. (*n* = 6).

The escape latency progressively decreased along the four days of training, showing improved acquisition behavior in all groups. The aluminum chloride (AlCl_3_) group presented a significant rise in escape latency to get to the hidden platform within the Morris Water Maze (MWM) as compared to the control group. Nonetheless, dapagliflozin significantly improved the escape latency in treated rats (*p* < 0.05). When compared to the control group, AlCl_3_-induced rats spent significantly less time in the target quadrant, while dapagliflozin increased the time spent in the target quadrant in all treated groups (*p* < 0.05) (Figure 1a,b).

#### 2.1.2. Dapagliflozin Effect on Y-Maze Spontaneous Alternation

**Figure 2:**
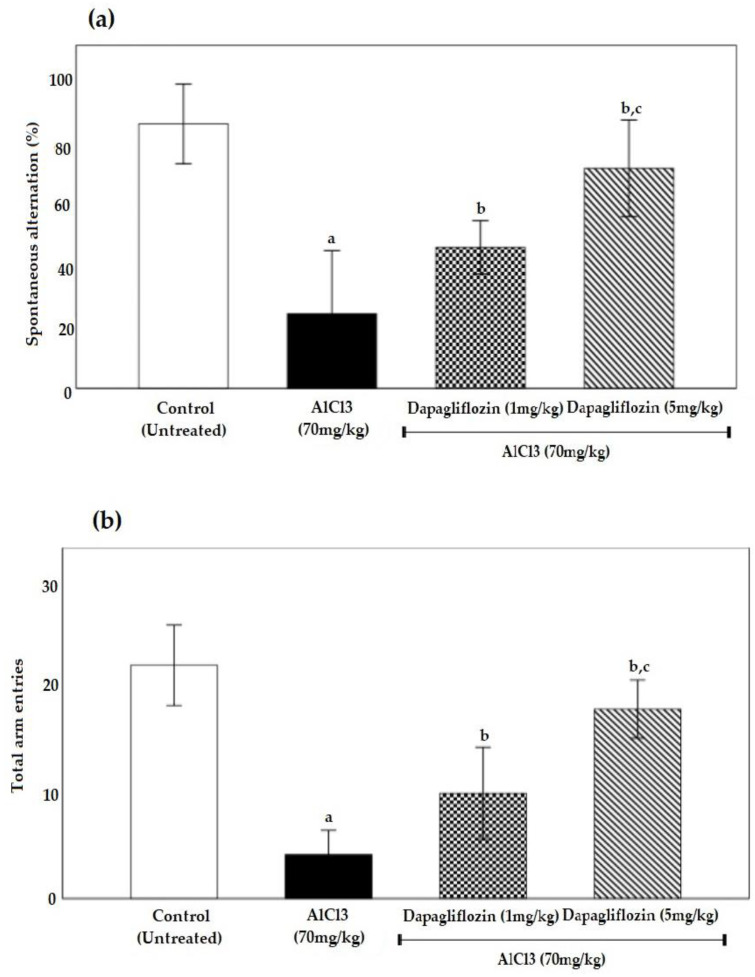
Effect of dapagliflozin (1 and 5 mg/kg) on (**a**) spontaneous alternation (%) and (**b**) total arm entries during the induction of AD. Data expressed as mean ± standard deviation and analyzed using one-way ANOVA followed by Tukey’s comparison tests. a: significant versus control group, b: significant versus AlCl_3_ (70 mg/kg), c: significant versus AlCl_3_ (70 mg/kg) + dapagliflozin (1 mg/kg). AD: Alzheimer’s disease, AlCl_3_: aluminum chloride. (*n* = 6).

The AlCl_3_ group was able to reduce the total entries and spontaneous alternation of the Y-maze spontaneous alternation (Y-maze) in comparison to the control group, resulting in a low percentage score. However, dapagliflozin therapy could increase the total entries and spontaneous alternation, resulting in a high percentage score (Figure 2a,b).

### 2.2. Biochemical Parameters

#### 2.2.1. Dapagliflozin Therapy Reduces Acetylcholinesterase

**Figure 3:**
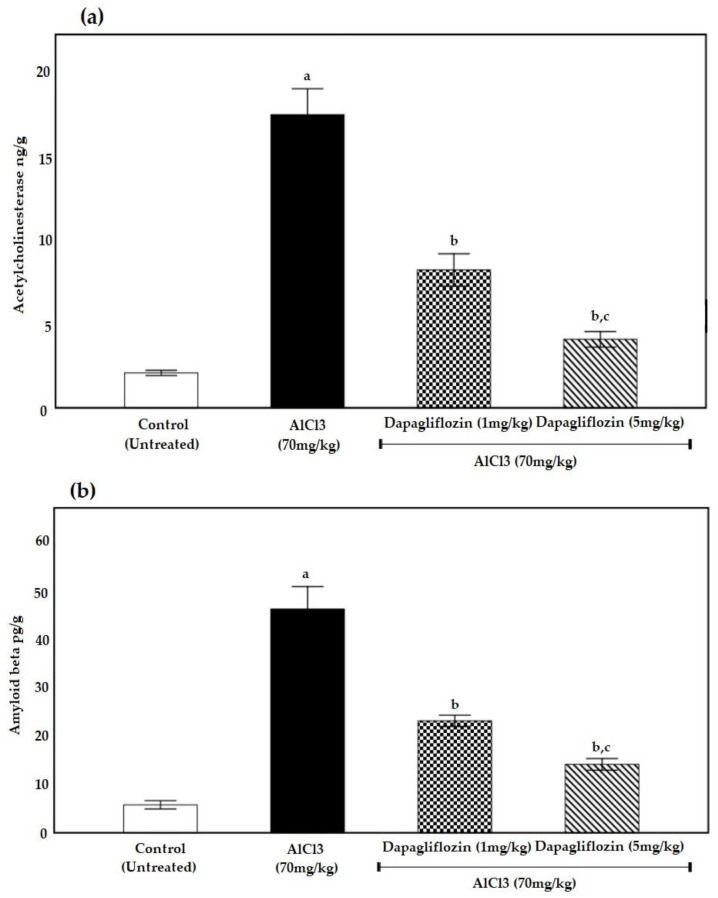
Effect of dapagliflozin (1 and 5 mg/kg) on (**a**) AChE and (**b**) Aβ during the induction of AD. Data expressed as mean ± standard deviation and analyzed using one-way ANOVA followed by Bonferroni’s post hoc comparison tests. a: significant versus control group, b: significant versus AlCl_3_ (70 mg/kg), c: significant versus AlCl_3_ (70 mg/kg) + dapagliflozin (1 mg/kg). AD: Alzheimer’s disease, AlCl_3_: aluminum chloride, AChE: acetylcholinesterase. (*n* = 6).

Compared to the control group, the AlCl_3_ group demonstrated a remarkable increase in AChE content. However, in a dose-dependent manner, dapagliflozin-treated groups showed a marked reduction in AChE activity as compared to the AlCl_3_ group at (*p* < 0.05) (Figure 3a).

#### 2.2.2. Dapagliflozin Suppresses Brain Amyloid β Protein Deposition

The levels of Aβ were measured in order to confirm the neuroprotective properties of dapagliflozin. When comparing the AlCl_3_-treated group to the control group, the levels of Aβ were considerably higher (*p* < 0.05). When compared to the AlCl_3_ group, dapagliflozin resulted in a substantial drop (*p* < 0.05) in Aβ levels in a dose-dependent way (Figure 3b).

#### 2.2.3. Dapagliflozin Inhibits Oxidative Stress Markers

Treatment with dapagliflozin resulted in a considerable decrease (*p* < 0.05) in malondialdehyde (MDA) levels as compared to the AlCl_3_ group, in which MDA levels were extremely high. Levels of superoxide dismutase (SOD), glutathione (GSH) and catalase (CAT) were extremely low in AlCl_3_-injected rats. In contrast to the AlCl_3_ group, dapagliflozin treatment showed a significant rise in SOD, GSH and CAT activities in a dose-dependent manner (Table 1).

### 2.3. Histopathological Examination of the Brain

#### 2.3.1. Dapagliflozin Effect on Cerebral Cortex

**Figure 4:**
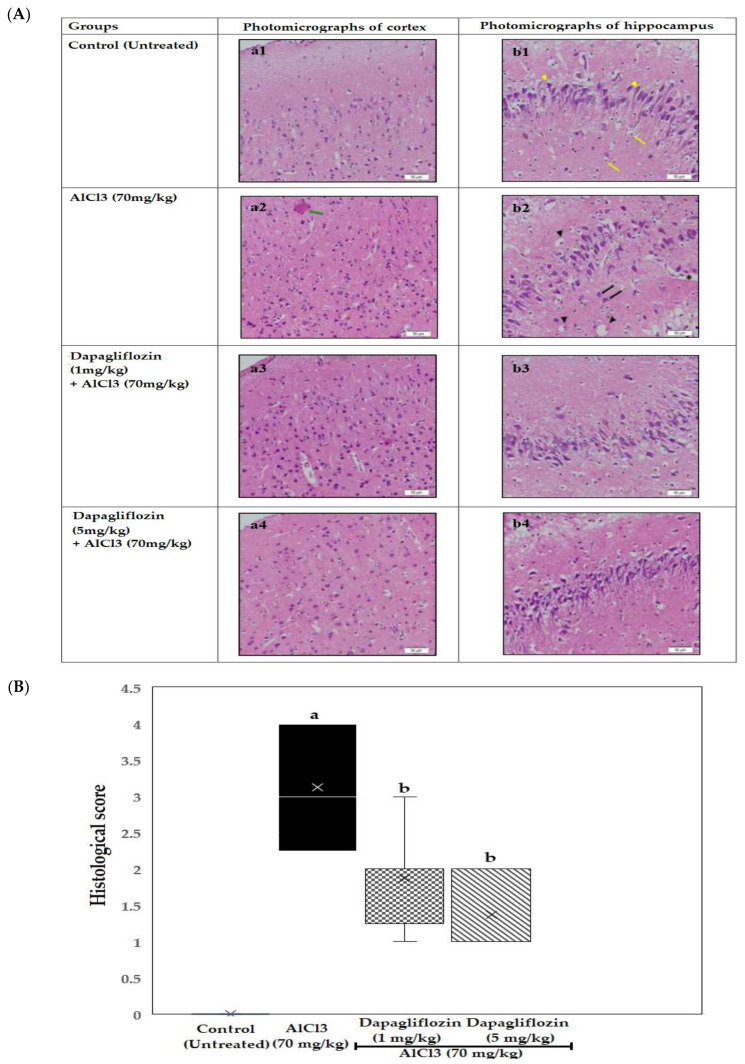
(**A**) Effect of dapagliflozin (1 and 5 mg/kg) treatment on (**A**) histopathological investigation of the brain during the induction of AD. (a1–4) Photomicrographs of cerebral cortex stained with H&E (X400) and (b1–4) Photomicrographs of hippocampus stained with H&E (X400). Examination showed remarkable AP (green arrow), normal structure of neurons (yellow arrow heads) and glial cells (yellow arrows), congested blood capillaries (black asterisk), pyknotic nuclei (black arrow head) and NFT (black arrow). AD: Alzheimer’s disease, AlCl_3_: aluminum chloride, H&E: hematoxylin and eosin, AP: amyloid plaques, NFT: neurofibrillary tangles. (*n* = 6). (**B**) Histopathological scoring. The severity of degeneration of neurons was scored from 0 to 5 as follows (absent, mild, moderate, severe, very severe, extremely damaged). Quantitative data were analyzed using one-way analysis of variance (ANOVA) after assessing the normality by Shapiro–Wilk test followed by Bonferroni’s post hoc multiple comparisons test. Histological scores are expressed as median and quartiles and analyzed by Kruskal–Wallis test followed by Dunn’s multiple comparison at *p* > 0.05. (*n* = 6).

Examination of the brain tissue of control untreated rats showed extremely active nerve cells, with large nuclei having faint nuclear chromatin. The adjacent inactive support cells had small nuclei with condensed chromatin and no visible nucleoli, revealing normal cerebral tissue. Sections of rat brains in the AlCl_3_ group displayed brain necrosis, plaques and the absence of normal structure and nuclei of cells. Dead nuclei appeared as dark NFT, amyloid plaques (APs) and congested blood capillaries. On the other hand, the dapagliflozin (1 mg/kg)-treated group showed significant improvement, with fewer pyknotic cells with less capillary congestion, tangles and plaques. The dapagliflozin (5 mg/kg)-treated group showed normal histological cellular structure (Figure 4(a1–a4)).

#### 2.3.2. Dapagliflozin Effect on Hippocampus

The control untreated group showed normal histo-architecture in the zones of the hippocampus; the molecular, granular and polyform layers. Both the neurons and glial cells were morphologically normal, with fine cellular distribution. The hippocampal tissues of the AlCl_3_ control group revealed marked degenerative changes of the glial cells and neurons, with cytoplasmic vacuolation. The examined tissues revealed marked multiple AP formation between cells with NFT. There was a marked decrease in the number of both neurons and glial cells, accompanied by an increased size and number of plaques. The dapagliflozin (1 mg/kg) group showed an improvement in the histological pattern of the hippocampus, with less (degenerated) AP between cells and reduced cytoplasmic vacuolation of neurons and glial cells. The examined tissues of the dapagliflozin (5 mg/kg) group showed normal histological architecture of the hippocampal tissue and cells (Figure 4(b1–b4)).

### 2.4. Dapagliflozin Alleviates Brain Glucose Levels

The AlCl_3_-treated rats demonstrated a significant rise in brain glucose levels compared to the control group. Treatment with dapagliflozin (1 and 5 mg/kg) diminished brain glucose levels compared to the AlCl_3_ group (Table 2).

### 2.5. Dapagliflozin Increases the Levels of Glucose Transporters and Glycolytic Enzymes

**Figure 5:**
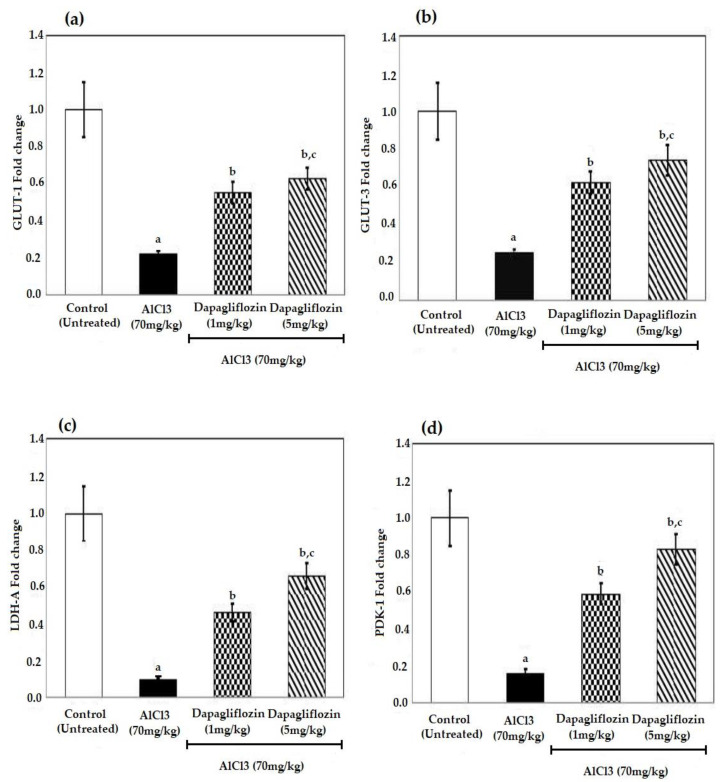
Effect of dapagliflozin (1 and 5 mg/kg) on (**a**) GLUT-1, (**b**) GLUT-3, (**c**) LDH-A and (**d**) PDK-1 during the induction of AD using PCR. Data expressed as mean ± standard deviation and analyzed using one-way ANOVA followed by Bonferroni’s post hoc comparison tests. a: significant versus control group, b: significant versus AlCl_3_ (70 mg/kg), c: significant versus AlCl_3_ (70 mg/kg) + dapagliflozin (1 mg/kg). AD: Alzheimer’s disease, AlCl_3_: aluminum chloride, GLUT-1: glucose transporters-1, GLUT-3: glucose transporters-3, LDH-A: lactate dehydrogenase-A, PDK-1: pyruvate dehydrogenase kinase-1. (*n* = 4).

The AlCl_3_ group revealed decreased expression of the glucose transporters (GLUT)-1,3, lactate dehydrogenase-A (LDH-A) and pyruvate dehydrogenase kinase-1 (PDK-1) compared to the control group (*p* < 0.05). Treatment with different doses of dapagliflozin induced a significant upregulation in both glucose transporters-1 (GLUT-1) and glucose transporters-3 (GLUT-3) when compared to the AlCl_3_ group at *p* < 0.05. Further, treatment with dapagliflozin significantly increased the expression of PDK-1 and LDH-A genes compared to the AlCl_3_ group at *p* < 0.05. The observed effect of dapagliflozin using Polymerase Chain Reaction (PCR) was dose dependent (Figure 5).

### 2.6. Dapagliflozin Upregulates Phosphorylated 5’ AMP-Activated Protein Kinase, Phosphorylated Mammalian Target of Rapamycin and Heme Oxygenase-1 Signaling Pathways

**Figure 6:**
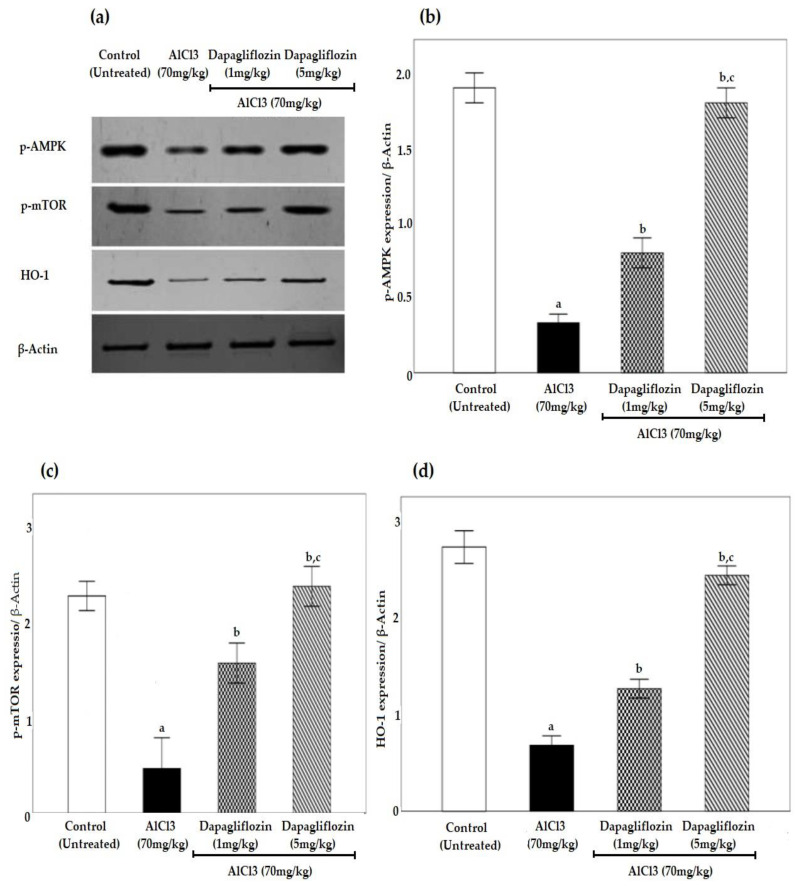
Effect of dapagliflozin (1 and 5 mg/kg) on (**a**) western blot of all groups with data normalized to β-actin, (**b**) p-AMPK, (**c**) p-mTOR and (**d**) HO-1 during the induction of AD. Data expressed as mean ± standard deviation and analyzed using one-way ANOVA followed by Bonferroni’s post hoc comparison tests. a: significant versus control group, b: significant versus AlCl_3_ (70 mg/kg), c: significant versus AlCl_3_ (70 mg/kg) + dapagliflozin (1 mg/kg). AD: Alzheimer’s disease, AlCl_3_: aluminum chloride, p-AMPK: phosphorylated 5’ AMP-activated protein kinase, p-mTOR: phosphorylated mammalian target of Rapamycin, HO-1: heme oxygenase-1. (*n* = 3).

Significant downregulation (*p* < 0.05) of phosphorylated 5’ AMP-activated protein kinase (p-AMPK), phosphorylated mammalian target of Rapamycin (p-mTOR) and heme oxygenase-1 (HO-1) was observed in the AlCl_3_ group compared to the control group. Dapagliflozin activated pAMPK, pmTOR and HO-1 signaling pathways in a dose-dependent way compared to the AlCl_3_ group using western blot analysis (Figure 6).

## 3. Discussion

Alzheimer’s disease is the most frequent neurodegenerative illness, with over 20 million cases worldwide [22], characterized initially by memory loss, followed by functional impairment in many areas of cognition [23]. In AD, neuropsychiatric comorbidities, such as depression, schizophrenia and bipolar disorders, are common [24]. Studies show that Al causes neuronal degeneration in the brain, leading to the generation of free radicals [25] that are responsible for neurotoxicity and damage of the neuronal membrane [26].

The current study showed that the time spent on reaching the platform was prolonged in the MWM task, indicating that learning abilities and spatial memory are impaired following chronic exposure to Al, as it accumulates in every part of the brain of rats, but mainly in the hippocampus, a center of memory and learning [27]. Moreover, the AlCl_3_ group had a lower alternation rate, demonstrating less sustained cognition and poor working memory in the Y-maze test. This concluding data could be due to deterioration in cognitive functions, such as learning, memory and retrieval abilities, evidenced by a low percentage alternation number of repeated entries into the same arm [28].

In the present study, changes in behavioral performance were accompanied by a remarkable increase in brain AChE levels in the AlCl_3_-administered groups. AlCl_3_ acts as a cholinotoxin, causing degradation of cholinergic terminals in the cortex and hippocampus, with subsequent neurodegeneration [29].

The peripheral anionic site of AChE has also been shown to form a stable complex with senile plaque components. There is also evidence that the presence of AChE increases amyloid neurotoxicity [30]. In addition, high levels of AChE cause a decrease in ACh, a neurotransmitter essential for processing memory and learning, supporting the cholinergic hypothesis of AD. On the other hand, studies have demonstrated a reduction in AChE caused by AD in humans [31] or aluminum in animal models of AD [32,33]. This controversy may be caused by a time-dependent impact, in which acute exposure to AlCl_3_ raises AChE activity, but chronic exposure decreases AChE activity.

The aluminum chloride group showed a rise in Aβ levels as compared to the control group, which matches with studies suggesting that Al plays a crucial role in the formation of Aβ deposits and fibrillary AP in AD [34].

The induction of AD by AlCl_3_ was confirmed by the histopathological alterations of the brain in the present study. These findings correlated with research indicating that, in the hippocampus, Al concentrations are highest, which makes it highly susceptible to neurofibrillary degeneration [35]. Axonal length and dendritic terminals are diminished in the hippocampus as a result of Al toxicity [36].

The AlCl_3_ group demonstrated a noticeable increase in the brain MDA, accompanied by a marked decrease in SOD, GSH and CAT, indicating a state of OS, which is linked to the pathogenesis of AD. This finding is in agreement with the effect of Al exposure on lipids, membrane-associated proteins and the activity of endogenous antioxidant enzymes during OS [37]. Neurons contain a large number of polyunsaturated fatty acids that react with ROS, resulting in lipid peroxidation and cellular damage [38].

Additionally, neurons contain low levels of GSH, an important antioxidant that neutralizes free radicals [39]. Therefore, neurons are highly susceptible to OS [40]. Another study also demonstrated that Al has damaging effects on the activities of glutathione reductase, glucose-6-phosphate dehydrogenase and LDH in blood. Glutathione reductase is an enzyme catalyzing the reduction of glutathione disulfide to GSH. This reaction is of vital importance since it protects the SH-groups of proteins from oxidation. This influence on enzyme activities was attributed to the interaction of Al with the -SH groups of the enzyme at the active site, hence stopping the sulfhydryl groups from working in some chemical reactions. The toxic effects of Al on enzyme activities may in part be due to its interference with mitochondria, thus impeding electron transport and the energy required for protein synthesis [41].

Considering that glucose is the main energetic fuel in brain tissue, glucose transport into brain cells through the BBB is vital for normal energy metabolism and physiological function [42]. Since neurons cannot make glucose, they rely entirely on glucose uptake through glucose transporters. In some diseases, such as AD, the reduced GLUT-1 and GLUT-3 levels may weaken the glucose-transporting capability, leading to reduced glucose uptake, and this impairment appears to be a cause of neurodegeneration [43].

In addition, glucose hypometabolism, caused by decreased glucose uptake, has been known as a key anomaly in the early stages of AD [44]. The expression of GLUT-1 and GLUT-3 is chiefly adjusted by hypoxia inducible factor-1 (HIF-1). Previous research has found that HIF-1, particularly HIF-1a, is reduced in the AD brain, suggesting that this reduction could be the source of GLUT-1 and GLUT-3 reduction. Oxidative stress, which is amplified in the AD brain, can disrupt HIF-1 [43].

In vitro studies have revealed that increased lipid peroxidation caused by Aβ deposition reduces glucose transport and metabolism in hippocampal and cortical neurons [45]. These outcomes are in line with the theory that diminished brain glucose uptake results in reduced glucose metabolism and abnormal tau phosphorylation and/or neurofibrillary degeneration [46]. It was found that GLUT-1 and GLUT-3 levels were significantly decreased in AD, thus enhancing brain glucose transport could be a probable therapeutic goal for treating AD [47].

Higher glucose buildup in the brain has been proven in AD patients, leading to hyperglycemia in the brain [48]. The BBB may adapt to prolonged hyperglycemic conditions and so limit glucose uptake into the brain as a protective strategy [49].

In this context, a decline in the production of LDH and PDK has been observed due to Al exposure, supporting the current results [50]. In earlier research, Al inhibited LDH activity in the cytosol of animals in a concentration-dependent manner at relatively high concentrations, proving that it is an effective inhibitor of brain glycolysis. It has also been shown that LDH and PDK are downregulated in a transgenic AD mouse model [51].

Overexpression of either the PDK or LDH enzymes in nerve cell lines inhibits mitochondrial respiration and imparts resistance to Aβ and other neurotoxins [51], supposing that these enzymes are necessary for defending cells against Aβ toxicity [52].

Regarding AD, LDH and PDK expression decreased, resulting in increased OxPhos, high ROS generation, greater Aβ sensitivity, mitochondrial dysfunction, elevated apoptosis, synaptic degradation, nerve cell death and ultimately cognitive impairment [53]. Mitochondrial dysfunction is a characteristic feature of AD, causing excessive ROS production, and it is thought to be at the core of Aβ toxicity [54].

The mTOR, composed of two complexes (mTORC1 and mTORC2), regulates protein translation and cell growth by responding to a variety of environmental variables, such as growth factors, nutrition and energy [55]; phosphatidylinositol 3-kinase (PI3K)/Akt (Protein Kinase B) and AMPK are the major regulators of mTOR [56]. In agreement with the findings of the present study, several studies have found a significant effect of Al exposure on the protein expression of PI3K, Akt and mTOR, which are involved in neuronal plasticity [57], implying that increased Al exposure in the brain is accompanied by decreased expression of the mTOR signaling pathway, leading to neuronal damage and cognitive decline [58].

The PI3K/Akt/mTOR signaling dysregulation can enhance glycogen synthase kinase-3beta (GSK-3beta) activity and cause tau hyperphosphorylation, resulting in NFT production [59]. This also illustrates why PI3K/Akt/mTOR signal inhibition is seen in the brains of Alzheimer’s patients. The major component of precipitated plaques, Aβ, interacts with the PI3K/Akt/mTOR pathway [60]. In neuronal cells and stem cells, Aβ causes neurotoxicity by blocking the PI3K/Akt/mTOR pathway. The GSK-3beta is largely triggered by Aβ oligomers, limiting PI3K/Akt/mTOR pathway activity [61].

The present study showed that p-AMPK decreased in the AlCl_3_ group. The AMPK is a principal regulator of cellular energy homeostasis and plays a vital role in glucose and lipid metabolism [62]. In the AD brain, AMPK activity declines [63], indicating decreased mitochondrial biogenesis and function [64]. Furthermore, AMPK is considered a critical participant in the deficiencies caused by Aβ oligomers [65]; hence, any decrease in its activity will have a negative impact on the AD brain. Moreover, it has been shown that AMPK activation is responsible for promoting glucose uptake and improving brain energy metabolism [66] because it regulates transport mechanisms and protects the cells from hypoxic damage [67]. It has been suggested that AMPK activation may be responsible for inhibiting beta-secretase 1 enzyme expression [66], enhancing the autophagic clearance of Aβ and aggregated tau [68], decreasing the activity of GSK-3beta and halting OS [65]. The effects mentioned above are totally opposite to what occurs in AD. However, a few studies have found that AD patients’ brain tissue AMPK activity (phosphorylation) was increased [68].

This study confirmed that HO-1 was decreased in the AlCl_3_ group. The HO-1 is an enzyme well known for its cytoprotective abilities in animals and plants. It provides various benefits against OS in different tissues via antioxidant, anti-apoptotic and anti-inflammatory mechanisms [69]. A striking inhibitory effect of APP on HO has been reported, where HO binds APP. Inhibition of HO could be neurotoxic by decreasing bilirubin formation and leading to less iron efflux from cells [70]. In contrast, a few studies have shown that HO-1 expression increased in AD brains compared to controls, suggesting this process might act as a safeguard against oxidative damage caused by Al treatments [71].

It has been suggested that SGLT2i may possess neuroprotective properties [72]. The reported side effects of dapagliflozin include hypotension, volume depletion, weight loss and increased risk of mycotic infection [73].

Dapagliflozin improved the disruption in memory function in the present study, as evidenced by a decrease in escape latency on different days of training and an increase in the time spent in the target quadrant as compared to the AlCl_3_ group in the MWM test, demonstrating mild improvement in learning and memory capabilities.

A high percentage alternation is characterized by a high percentage of entries into consecutive arms. As long as the animal has a strong working memory, it will remember which arms of the maze it has already explored and will tend to enter less recently explored arms that require communication between several brain regions, such as the hippocampus and prefrontal cortex [74]. The findings of the current study indicate that dapagliflozin treatment restored normal function, which, in turn, was reflected in the neurological scoring. This improvement could be due to memory enhancement through inhibition of AChE activities in brain areas [75]. The same discrepancy has been observed with SGLT2i treatment, where it positively affected cognitive abilities and significantly ameliorated memory impairment [72].

Dapagliflozin was also able to repair the disturbance in neurotransmission in the present study, showing decreased AChE activity as compared to the AlCl_3_ group. Dapagliflozin may have a neuroprotective effect by conserving synaptic plasticity, insulin resistance and stopping the decline in cognition [76]. It may also inhibit AChE; thus, it was considered as a hopeful drug for the management of diabetes-related neurological disorders [77].

A previous study recorded AChE inhibition by another SGLT2i oral treatment, which agrees with the current results. The findings appear to be in line with molecular docking approach studies examining the binding energy of dapagliflozin and the 19 amino acid residues of AChE [72]. Hence, dapagliflozin could be a powerful dual inhibitor of SGLT2 and AChE [77].

A study regarding a similar SGLT2i reported decreased senile plaque density and a general decrease in soluble and insoluble Aβ levels in the cortex and hippocampus of the treated model. Based on their antioxidant and anti-inflammatory properties, the SGLT2is not only improve brain mitochondrial function and insulin signaling, but also reduce cell death. Furthermore, SGLT2is prevent cognitive decline and protect synaptic plasticity in the hippocampus [76].

It has been reported that SGLT2is decreased the expression of caspase-3. Take into consideration that caspase-3 overproduction may be involved in AD pathogenesis including amyloidosis [72]. These results agree with the recent study where dapagliflozin treatment reduced the levels of Aβ, displaying that dapagliflozin is effective in avoiding the deposition of phosphorylated tau and Aβ in rats with AD.

It has been found that SGLT2is can stimulate multiple antioxidant, anti-inflammatory and anti-apoptotic signaling pathways. The amelioration of cognitive performance by SGLT2is appears to be due to the reduction of OS [78].

This agrees with previous work that reported antioxidant effects of dapagliflozin treatment significantly reduced levels of MDA and increased levels of antioxidants compared to their corresponding control. These data indicate that dapagliflozin has a remarkable antioxidant action [79].

Glucose transporter 1 is present in the BBB and glial cells, while GLUT-3 is present in neurons [80,81]. Studies have shown SGLT2i directly induces upregulation of GLUT-1 expression in cardio-myocytes. Moreover, increased SGLT2i-dependent GLUT-1 expression enhanced glucose uptake and the intracellular glucose concentration [82]. As mentioned earlier, AD decreases the expression of GLUT. Using GLUT as a target could have a favorable impact. High GLUT-1 and GLUT-3 levels that are observed with dapagliflozin treatment in the current study may improve the glucose transporting ability, resulting in increased glucose metabolism and absorption.

In the present study, dapagliflozin increased the levels of LDH-A and PDK-1. This diminished mitochondrial activity and caused resistance to Aβ [51]. These results agree with a study showing that the administration of SGLT2i reestablished the elevated levels of LDH, which may be a result of ameliorating insulin resistance and the counteraction of elevated levels of inflammatory cytokines [83]. Interestingly, it has been found that Aβ-resistant nerve cells display a shift in metabolism to favor lactate production. This switch in metabolism from mitochondrial respiration to glycolysis is driven in part by HIF-1𝛼 [53]. The HIF-1𝛼 has an influence on shutting down mitochondrial respiration by encouraging PDK-1 transcription and increasing expression of GLUT and glycolytic enzymes, thereby increasing the conversion of glucose to pyruvate. Additionally, HIF-1 induces the transcription of LDH-A, increasing the conversion of pyruvate to lactate. This mitigates mitochondrial ROS production, making cells more resistant to apoptosis in the presence of Aβ [53].

Dapagliflozin has been shown to activate AMPK and mTOR, increase the Rapamycin-insensitive companion target of Rapamycin (RICTOR) levels and increase the interaction of mTOR with RICTOR. Dapagliflozin increased the phosphorylation of Akt, proposing special activation of mTORC2, preferring glucose uptake, glycolysis and cell survival [49]. These results are in line with the present study, where dapagliflozin raised p-mTOR levels in treated groups.

The SGLT2is have been reported to activate AMPK. It has been further shown that SGLT2is augmented antioxidant/anti-inflammatory signaling that involves AMPK, Akt, endothelial nitric oxide synthase, nuclear factor-erythroid factor 2-related factor2 (Nrf2) and HO-1. It was proved that SGLT2i treatment significantly improved phosphorylation and activation of AMPK [84] to produce a range of useful effects in the cardiovascular system and beyond.

It has been established that SGLT2i enhances cardiomyocyte AMPK to stimulate autophagy, leading to improvement in heart function via enhancing cardiac energy metabolism [85]. Activating AMPK can help to enhance the brain’s energy metabolism, which is important in the etiology of AD. The activation of AMPK can help reduce Aβ production by modulating APP processing. Thus, AMPK appears to be a neuroprotective factor against metabolic stress, suggesting that it may play a role in the prevention of AD pathogenesis [64].

The relation between phosphorylation of AMPK and mTOR is controversial. It was reported that amino acids activate AMPK concurrently with mTOR [86]. However, another study showed that dapagliflozin enhanced AMPK and reduced mTOR [20]. This may be attributed to the different animal models, as the current study used AlCl_3_-induced AD, while the other study used a D-galactose Alzheimer’s rat model.

Dapagliflozin was observed to raise the levels of HO-1 in the rat brain, according to the current research. Studies found that SGLT2i increased Nrf2 protein levels; consequently, Nrf2 activation is required for HO-1 gene transcription to occur [87]. Similarly, canagliflozin therapy raised HO-1 levels and lowered OS in isoprenaline-treated rats [79]. 

Study limitation: The current biochemical determinations were performed on the whole brain homogenate instead of separating the hippocampus.

## 4. Material and Methods

### 4.1. Animals

Experiments were performed on 40 male albino Swiss rats, weighing (220–250 g). They were purchased from Nile Co. for Pharmaceuticals and Chemical Industries (Cairo, Egypt). The animals were divided randomly in cages under a controlled temperature (25 ± 4 °C), a regular light/dark cycle and unlimited access to food and water (ad libitum). Prior to the trial, rats were subjected to an adaptation period of seven days. The Suez Canal University, Faculty of Pharmacy’s ethical committee authorized the study in November, 2021with liscence no 202011MA. The *Guide for the Care and Use of Laboratory Animals* was followed in the operation of all experimental methods [88].

### 4.2. Drugs and Chemicals

The hydrated form of aluminum chloride (AlCl_3_.6H2O) was purchased from Sigma Chemical Co. (St. Louis, MO, USA) and was freshly dissolved in distilled water. Dapagliflozin was obtained from AstraZeneca, “Egypt Pharmaceuticals, New Cairo, Egypt”, and a fresh solution was prepared by dissolving it in 1% saline.

### 4.3. Induction of AD

For the induction of AD, AlCl_3_ was freshly prepared by dissolving it in distilled water and was given to rats with a single daily dose of (70 mg/kg, IP) [89] for 9 weeks. The LD50 for aluminum chloride in rats was 1283.7 mg/kg [90]. Pharmacologic interventions were initiated in week 6 and continued for 28 days, along with AlCl_3_. Learning and memory abilities were investigated using two well-documented tests, the MWM task and the Y-maze task.

### 4.4. Experimental Design

**Figure 7:**
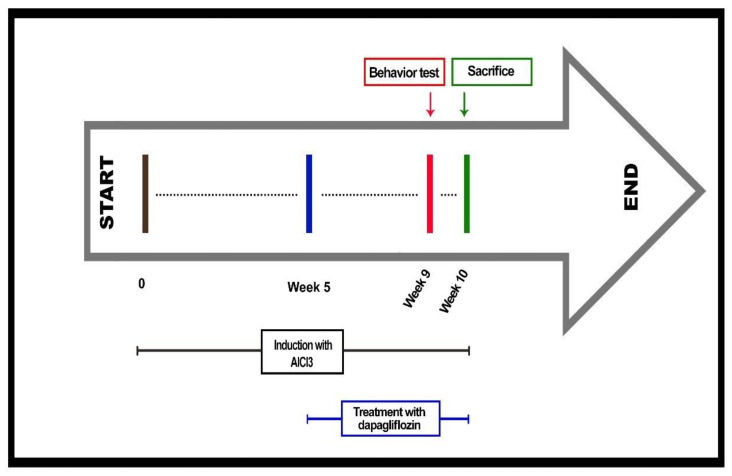
Schematic illustration of the present study.

Rats were randomly distributed into four groups: the first group, the control (untreated) group, received saline (10 mL/kg, IP); the second group, the AlCl_3_ group, received AlCl_3_ (70 mg/kg, IP) daily for 9 weeks. The third and fourth groups received AlCl_3_ (70 mg/kg, IP) daily for 5 weeks. Then, daily treatment was commenced with dapagliflozin (1 mg/kg PO) [76] and dapagliflozin (5 mg/kg PO) [83], along with AlCl_3_ for 4 weeks (Figure 7). The LD50 for dapagliflozin in rats was 1200–3000 mg/kg/day [91].

### 4.5. Sample Preparation

Approximately 24 h after the last test, rats were sacrificed under thiopental (40 mg/kg), and their whole brains were separated and washed in cold saline. Brain tissues were used for histopathological examination. Additionally, the remaining were kept at −80 °C for further analysis.

Brain samples were homogenized in saline for the assessment of brain glucose levels, OS markers, AChE activity and Aβ peptide. The remaining samples were used for western blot analysis of p-AMPK, p-mTOR and HO-1, and real-time PCR was used to examine GLUT-1, GLUT-3, LDH-A and PDK-1.

### 4.6. Behavioral Experiment

The rats were transported to the test location for adaptation an hour prior to each trial, with food and water removed from the cages. There was a particular time limit for all experiments to be conducted (from 8 AM to 6 PM). To formulate an integrative test pattern, two different experiments of behavioral assessments with varying stages of stress were selected. The chosen tests yielded the best measures of the behavioral responses to the drug.

#### 4.6.1. Morris Water Maze Task

In this experiment, a 150 × 62.5 cm black circular tank serves as the water maze; rats are taught to use visual cues around the maze to locate an escape platform. Water was added to the tank to a depth of 40 cm and heated to room temperature during the experiment to maintain the temperature at (23–30 + 1 °C). Additionally, placed around the room were numerous visual cues. The maze was divided into four quadrants: northeast (NE), northwest (NW), southeast (SE) and southwest (SW), with beginning points north (N), south (S), east (E) and west (W). During four consecutive daily sessions, rats were trained to discover the submerged (1 cm) escape platform (diameter: 13 cm) that was in a fixed position in the center of the SW quadrant, where the pool of water was colored opaque with powdered nonfat milk. The rats’ swimming patterns were recorded using a video tracking camera. Four trials of each session were conducted, using four different starting locations in a predetermined order.

To ensure complete recognition, the rats were allowed to remain on the platform for 20 s at the end of each session, and if they were unable to locate the platform within 60 s, they were placed on it for 10 s. Training trials were averaged for escape latency (four different positions), and the averages of group results were calculated. In the final training trial (two hours after the fourth trial on the fourth day), the rats were subjected to a memory probe trial, in which they were required, in the absence of the training platform, to swim for 60 s. The rats all started in the same position, on the opposite side of the target quadrant. The probe trial time was measured in seconds spent in the target quadrant [92].

#### 4.6.2. Y-Maze Spontaneous Alternation

Galvanized 20-gauge sheet metal was used to build the maze. The three arms, A, B and C, were each 50.8 cm long and 17.7 cm wide, and they were all angled at 120°. Animals were placed just within arm B, away from the center for the test, and they were free to move around the apparatus for eight minutes. The rats were recorded using a video tracking camera set above the maze. An entrance is defined as when all four of the rat’s limbs are within one arm, while consecutive entries into all three arms are described as an alternation. A stopwatch was used to record the number of entries (one for each arm). Then, the number of alterations (a set of 3 consecutive letters) was divided by the total number of sets of 3 letters (a set of 3 letters) × 100 [93].

### 4.7. Biochemical Parameters

#### Determination of AChE and Aβ Level

In the brain tissue homogenate, AChE (Rat AChE enzyme-linked immunoassay (ELISA) kit catalog no. E-EL-R0355, Elabscience, Inc., Houston, Texas, USA) and Aβ (catalog no. MBS702915, MyBioSource, Inc., San Diego, CA, USA) activities were estimated by the Quantitative Sandwich ELISA technique, as specified by the instructions of the manufacturer.

### 4.8. Assessment of Brain OS Markers

The level of MDA, a byproduct of lipid peroxidation, was measured with the Competitive ELISA technique (Rat MDA ELISA kit catalog no. LS-F28018, LSBio, Seattle, WA, USA). The levels of SOD (Rat SOD ELISA kit catalog no. MBS036924, My BioSource, Inc., San Diego, CA, USA), GSH (Rat reduced GSH ELISA kit catalog no. E02G0367, ShangHai, BlueGene Biotech CO., Ltd., Shanghai, China) and CAT (Rat CAT ELISA kit catalog no. MBS006963, MyBioSource, Inc., San Diego, CA, USA) were measured by Quantitative Sandwich ELISA technique kits, as stated by the manufacturer’s instructions.

### 4.9. Histopathological Investigation of the Brain

The brains were embedded with paraffin wax after being preserved in a 10% paraformaldehyde solution overnight. Hippocampal and cortical specimens were segmented, and 10% paraffin blocks were created. Hematoxylin and eosin (H&E) was used to routinely stain sections from prepared paraffin blocks (4 µm thick), which were then blindly evaluated.

### 4.10. Brain Glucose Levels

In accordance with the manufacturer’s instructions, levels of glucose in brain tissue were assessed (GOD/PAP) with a colorimetric kit (catalog no. 11801, Vitro Scient, Cairo, Egypt).

### 4.11. Quantitative Polymerase Chain Reaction Assay

The quantitative real-time PCR assay was used for the examination of the following: GLUT-1, GLUT-3, LDH-A and PDK-1. SV total RNA isolation technology was used to extract total RNA from both frozen and fresh brain tissue (4 different samples per group) (Promega, Madison, WI, USA). Using a NanoDrop spectrophotometer, the isolated RNAs concentration and purity were evaluated (Thermo Fisher Scientific, USA). Examination of the expression of the GLUT-1, GLUT-3, LDH-A and PDK-1 genes in brain tissue was performed using the GoTaq^®^ 1-Step RT-qPCR System (Promega, Madison, WI, USA). The endogenous control employed was glyceraldehyde 3-phosphate dehydrogenase (GAPDH). The annealing temperatures and primers are mentioned in (Table 3). The following materials were used in the experiment: 4 µL RNA template, 0.4 L GoScript^TM^ RT mix for 1-step RT-qPCR, 1 µL forward and reverse primers, 10 µL GoTaq^®^ qPCR master mix, 0.31 µL additional CXR reference dye, 3.29 µL nuclease-free water. The cycle included 15 min of reverse transcription at 37 °C, 10 min of reverse transcriptase enzyme inactivation at 95 °C, and 40 cycles of denaturation at 95 °C for 10 s, annealing for 30 s and extension at 72 °C for 30 s. The real-time PCR studies were carried out using the StepOnePlus^TM^ Real-Time PCR thermal cycling instrument (Applied Biosystems, Carlsbad, CA, USA). For each sample, the reaction was run in duplicate to ensure the results’ validity, and each run included a PCR negative control with no template to ensure no DNA contamination. Fold change was calculated as 2^−ΔΔCt^, with ΔΔCt and fold change calculated [94].

### 4.12. Western Blot

The ReadyPrepTM protein extraction kit from Bio-Rad Inc. (catalogue no. 163-2086) was applied in accordance with the manufacturer’s instructions. To preserve the integrity of the proteins and biological activity, the brain tissues were homogenized in ice-cold RIPA lysis solution containing protease and phosphatase inhibitors. The lysates were then centrifuged for 10 min, and the supernatants were stored at −80 °C. The Bradford assay was used [95] to determine protein concentrations (SK3041, Markham Ontario L3R 8T4 CanadaEqual); parts of the lysate and 2 Laemmli sample buffer (4% sodium dodecyl sulphate, 0.125 M tris hydrochloride, 20% glycerol, 10% 2-mercaptoethanol, 0.004% bromophenol blue [pH 6.8]) were combined. The mixture was then boiled at 95 °C for 5 min to ensure protein denaturation, mixed thoroughly for 30 s and centrifuged at 10,000× *g* for 10 min. The supernatants were then electrophoresed on a 12% sodium dodecyl sulfate–polyacrylamide gel. Proteins were transferred to PVDF membranes using Bio-Rad Trans-Blot Turbo technology (Bio-Rad Laboratories Ltd., Watford, UK) and then blocked for an hour in TBS containing 5% (wt/vol) nonfat dried milk and 0.05 percent polyoxyethylenesorbitan monolaurate (Tween 20). The prepared membranes were treated overnight at 4 °C with a rabbit monoclonal antibody (1:1000 dilution) as the primary antibody, followed by washing and incubation with the HRP-conjugated secondary antibody. The ChemiDoc MP imager was used to visualize the signals (catalog no. 12003154, Bio-Rad, Redmond, WA, USA).

### 4.13. Statistical Analysis

The mean and ± standard deviation were used to express all results. Following a one-way repeated measures ANOVA, the results were evaluated using Tukey’s (for behavioral tests) or Bonferroni’s post hoc comparison testing (for biochemical measurements). Statistical Package for the Social Sciences, version 26, was used to analyze the data (SPSS Software, SPSS Inc., Chicago, IL, USA). All relevant comparisons among the research groups were conducted, and a *p* value of < 0.05 was regarded as statistically significant.

Quantitative data were analyzed using one-way analysis of variance (ANOVA) after assessing the normality with the Shapiro–Wilk test, followed by Bonferroni’s post hoc multiple comparisons test.

Histological scores are expressed as median and quartiles and analyzed by the Kruskal–Wallis test, followed by Dunn’s multiple comparison at *p* < 0.05.

## 5. Conclusions

This study was conducted to evaluate the effect of dapagliflozin on cognitive decline associated with AD and to investigate the efficacy of dapagliflozin in inhibiting the amyloidogenic processing in AD. The current data demonstrated that dapagliflozin ameliorated features of AD induced by AlCl_3_ in rats, proving its neuroprotective effects by reportedly improving cognition. This improvement in learning and memory abilities using two well-known behavior tests, along with histopathological alterations, was mediated through different mechanisms: (1) Decreasing AChE levels; (2) Reducing Aβ accumulation; (3) Attenuating OS markers, such as MDA, SOD, CAT and GSH; (4) Increasing blood glucose levels; (5) Enhancing glucose metabolism by having a protective effect on glycolytic enzymes, as well as GLUTs; (6) Activating AMPK and mTOR signaling; (7) Increasing HO-1. 

## Figures and Tables

**Table 1:** Impact of dapagliflozin (1 and 5 mg/kg) treatment on brain OS biomarkers during the induction of AD. Data expressed as mean ± standard deviation and analyzed using one-way ANOVA followed by Bonferroni’s post hoc comparison tests. ^a^: significant versus control group, ^b^: significant versus AlCl_3_ (70 mg/kg), ^c^: significant versus AlCl_3_ (70 mg/kg) + dapagliflozin (1 mg/kg). AD: Alzheimer’s disease, AlCl_3_: aluminum chloride, OS: oxidative stress. (*n* = 6).

Groups	MDA ng/g	SOD u/g	GSH pg/g	CAT u/g
**Control (untreated)**	3.1 ± 0.2	41.2 ± 3.4	52.7 ± 2.4	37.5 ± 2.3
**AlCl_3_ (70 mg/kg)**	20.6 ± 1.9 ^a^	8 ± 1 ^a^	8.6 ± 1.1 ^a^	7.1 ± 0.8 ^a^
**AlCl_3_ (70 mg/kg) + Dapagliflozin (1 mg/kg)**	10.2 ± 1 ^b^	15.9 ± 1.7 ^b^	21.7 ± 1.3 ^b^	14.7 ± 1.6 ^b^
**AlCl_3_ (70 mg/kg) + Dapagliflozin (5 mg/kg)**	5.6 ± 0.6 ^b,c^	27.1 ± 0.5 ^b,c^	39.3 ± 0.8 ^b,c^	24.9 ± 1.4 ^b,c^

**Table 2:** Results of dapagliflozin (1 and 5 mg/kg) on brain glucose levels during the induction of AD by AlCl_3_ in rats. Data expressed as mean ± standard deviation and analyzed using one-way ANOVA followed by Bonferroni’s post hoc comparison tests. ^a^: significant versus control group, ^b^: significant versus AlCl_3_ (70 mg/kg), ^c^: significant versus AlCl_3_ (70 mg/kg) + dapagliflozin (1 mg/kg). AD: Alzheimer’s disease, AlCl_3_: aluminum chloride. (*n* = 6).

Groups	Brain Glucose Level mg/dL
**Control (untreated)**	13.7 ± 0.1
**AlCl_3_ (70 mg/kg)**	23.54 ± 1 ^a^
**AlCl_3_ (70 mg/kg) + Dapagliflozin (1 mg/kg)**	18.6 ± 0.5 ^b^
**AlCl_3_ (70 mg/kg) + Dapagliflozin (5 mg/kg)**	15.5 ± 0.2 ^b,c^

**Table 3:** Real-time PCR primers and annealing temperatures.

GenBank^®^ Accession No.	Gene	Primers	Annealing Temperature
**NM_138827.2**	GLUT-1	Forward: 5′-TGGCCAAGGACACACGAATACTGA-3′ Reverse: 5′-TGGAAGAGACAGGAATGGGCGAAT-3′	58 °C
**NM_017102.2**	GLUT-3	Forward: 5′-TGGCTACAACACCGGAGTCATCAA-3′ Reverse: 5′-CTGCCAAAGCGGTTGACAAAGAGT-3′	58 °C
**NM_017025.2**	LDH-A	Forward: 5′-GATCTCGCGCACGCTACT-3′ Reverse: 5′-CACAATCAGCTGGTCCTTGAG-3′	53 °C
**NM_053826.2**	PDK-1	Forward: 5′-TCCCCCGATTCAGGTTCAC-3′ Reverse: 5′-CCCGGTCACTCATCTTCACA-3′	54 °C
**NM_017008.4**	GAPDH	Forward: 5′-ATGACTCTACCCACGGCAAG-3′ Reverse: 5′-GATCTCGCTCCTGGAAGATG-3′	52 °C

## Data Availability

Data are available within the article.
